# Relationship between digital capabilities and academic performance: the mediating effect of self-efficacy

**DOI:** 10.1186/s12912-023-01593-2

**Published:** 2023-11-17

**Authors:** Rasha Kadri Ibrahim, Aisha Namshan Aldawsari

**Affiliations:** 1 Nursing Department, Fatima College of Health Sciences, Baynunah Complex, Al Dhafra Region, Madinat Zayed UAE; 2 Nursing Department, Faculty of Nursing, Damnhour University, Damanhour, Egypt; 3 Nursing Department, Fatima College of Health Sciences, Al Ain, UAE

**Keywords:** Academic performance, Digital capabilities, Self-efficacy, Digital literacy, Nursing students

## Abstract

**Aims:**

To assess digital capabilities and academic performance among nursing students and investigate the mediating role of students’ self-efficacy.

**Background:**

In the context of education and technology, digital capabilities, self-efficacy, and academic performance among nursing students are interconnected concepts. Students who use their digital capabilities and competencies combined with their belief in the ability to efficiently perform learning tasks could improve their academic endeavors. Nevertheless, insufficient consideration has been placed on research understanding of the mediating roles and broad elements that influence their relationships.

**Methods:**

A cross-sectional, correlational, descriptive, and quantitative study was established. During the 2022–23 academic year, data were gathered from 200 students. The Hayes Process Model 4 macro was employed to investigate the role of students’ self-efficacy mediating effect on the association between digital capabilities and academic performance.

**Results:**

The digital capability level and self-efficacy level were high. Additionally, the academic performance level was moderate. The mediation analysis revealed that the direct effect of digital capabilities on student performance in the presence of the mediator was significant (*b* = 0.0063, *p* = 0.022). Hence, self-efficacy partially mediated the relationship between digital capabilities and student performance.

**Conclusion:**

The study emphasized the importance of improving students’ digital capabilities that enhance their confidence and self-actualization. In addition, nursing students are encouraged to improve their sense of self-efficacy throughout their tenure in college because it is a predictor of future success.

## Introduction

In the contemporary digital age, nursing students are required to possess a set of essential competencies referred to as digital capabilities [1]. Digital capabilities include a variety of skills and knowledge related to the use of digital technologies, tools, and resources [2]. These competencies encompass proficiencies in digital independent learning, digital information/data management, digital communication and collaboration, digital creation, and digital problem-solving [2, 3]. These capabilities empower nursing students to deliver proficient and effective care while also being able to adapt to the ever-changing digital environment within healthcare institutions [1, 3].

The concept of digital capabilities is strongly associated with self-efficacy, particularly in the field of learning [4]. The acquisition and application of digital capabilities are influenced by students’ self-efficacy beliefs. When students have a strong belief in their capacity to acquire the necessary skills to effectively use digital technologies, they actively participate in the educational experience with enthusiasm and tenacity [5]. Conversely, students with low self-efficacy features in digital abilities tend to lose their path and fail to accomplish tasks when confronted with digital activities [6].

Academic performance is influenced by digital self-efficacy [7]. Students’ academic performance improves when they have trust in their digital skills and abilities [8, 9] Academic performance measures the extent to which intended learning outcomes have been attained and is recorded in a student’s cumulative grade point average (CGPA) [10]. Farrington et al. [11] described academic performance as a “complex phenomenon” in which “cognitive and noncognitive elements continuously interact in crucial ways to generate learning”.

The literature provides an understanding of the elements that may influence the academic performance of learners [8, 12]. A recent study reported that students’ academic performance was negatively affected during COVID-19, as the majority of classes were held online because students had difficulty navigating the online learning platform [13]. The authors additionally suggested devoting special attention to students’ digital abilities to ensure that all students have access to an equitable education [13]. Academic performance should be studied from a multifactor viewpoint. This study will assess the effect of students’ self-efficacy on the relationship between digital capabilities and academic performance.

## Background

### Digital capabilities

Digital capabilities in the realm of educational organizations are defined as “the skills, talents, and abilities that enable an individual to live, study, and operate in a digital society” [14–17]. Additionally, the concept of digital capabilities refers to how effectively students can employ critical thinking and problem-solving skills when working with digital tools and resources [18, 19]. Digital literacy, on the other hand, is a concept frequently used in the digital health care literature, which is related to digitalization, but it focuses on the skills used to locate and cite information [20, 21]. According to Gilster [22], digital literacy is “the capacity to comprehend and utilize information in diverse formats from a variety of sources when it is displayed on a computer”. It is crucial to differentiate between concepts that are related to digitalization to be able to identify the needs of students [23].

Thus, digital capabilities frameworks have been established to empower students as well as faculty to cope with the demands of digital capabilities [3, 24]. Among these frameworks is the Joint Information System Committee (JISC), which is a framework designed to encourage the use of digital technology in education [23, 25]. The JISC framework incorporates capabilities and enablers into six domains [3, 26]. The model’s primary focus is on digital proficiency and productivity, with the other five categories listed as digital creation, problem-solving, and innovation; digital learning and development; digital identity and well-being; information, data, and media literacies; and digital communication, collaboration, and participation [27, 28].

College students with strong digital capabilities may find it easier to navigate online courses, interact with digital learning platforms, and engage in virtual classroom activities [27]. There is a common assumption that students master high digital skills because they are considered the digitalized generation [15, 29]. This poses the question of whether these students have the digital capabilities to be able to locate, evaluate and, more importantly, analyze information [20]. A research study of nursing students in the UAE was conducted to examine nursing students’ digital capabilities, and the authors reported that while participating students had excellent digital literacy skills, they did not appear to be competent in analyzing such information [30]. As a result, educational institutions must ensure that students use critical thinking skills in their interactions with the digital world [19]. This argument is also emphasized in a study that investigated the level of digital competences among nursing students [31]. Learning about digital skills is a topic of prime concern in university education, and its influence on student performance has recently sparked substantial attention [20, 30].

### Self-efficacy

Bandura [32] defined self-efficacy as “the belief in one’s capacity to organize and execute the courses of action necessary to achieve specified goals” [32, 33]. Self-efficacy was described by Akhtar [34] as the confidence we have in our talent, particularly our capacity to face challenges and finish a task effectively [35]. It enables students to excel through their dedication and perseverance to complete their learning-related responsibilities [36, 37].

Self-efficacy is one of the most important determinants of academic achievement among nursing students [38]. Academic self-efficacy (ASE) pertains to students’ views and attitudes on their capacity to achieve academic achievement, as well as their ability to successfully complete academic activities [39–41]. It has been postulated that students who demonstrate high self-efficacy make efforts to find creative ways to approach challenges and solve problems; thus, self-efficacy could impact task selection and achievement [33, 42, 43].

Self-efficacy in relation to academic performance has received much attention because it appears to be the most influential factor in academic achievement [37]. Several researchers have examined self-efficacy and revealed that it has a major influence on student learning, motivation, and academic functioning [41, 44, 45]. In more recent research performed at three Chinese institutions, researchers investigated the learning situation of preservice special education teachers and observed that their ASE was only at a medium level [41].

The self-efficacy of nursing college students was linked to their digital skills, which was an important variable in academic learning [46]. For example, a study assessed the relationship between self-efficacy toward online eHealth literacy levels among nursing students [47]. The author concluded that there was a significant relationship between self-efficacy and the students’ use of eHealth [47]. Another recent study conducted at three nursing colleges in Korea to identify the relationship and direction of factors affecting nursing students’ Ehealth literacy in an online learning environment indicated that digital literacy and self-efficacy were found to be associated with Ehealth literacy and mediate the relationship between online learning attitude [37].

### Academic performance (AP)

Academic performance could be improved by integrating digital capabilities and fostering self-efficacy [48]. The interplay of these factors can significantly influence a student’s academic success. Students’ grades are generally used as a proxy for their academic progress when examining the effects of educational methods [37]. A high-grade point average in college is a reliable sign of academic success [49].

Some studies have found evidence of a positive relationship between technology and student learning in terms of digital abilities [49, 50]. Students who possess varied sets of digital abilities and are skilled with technology often demonstrate good academic performance [51, 52]. A study conducted at a Korean college with a total of 614 undergraduates to determine the relationship between university students’ experience in e-learning and academic achievement (GPA) revealed that university e-learning settings need students to have strong digital skills to perform academic work and a commitment to active participation in the environment of academic learning [49].

A close association between self-efficacy and student academic (GPA) performance has been noted in educational settings since self-efficacy increases students’ perception of accomplishment and thus helps them achieve superior academic performance [53–55]. In the nursing literature, current research has shown that students who believe in their own abilities to successfully complete academic tasks perform better academically [37, 54, 56]. In 2021, Reynolds et al. conducted a systematic review that aimed to explore the relationship between noncognitive factors, including self-efficacy. It was found that high self-efficacy is associated with better performance [57].

**Fig. 1:**
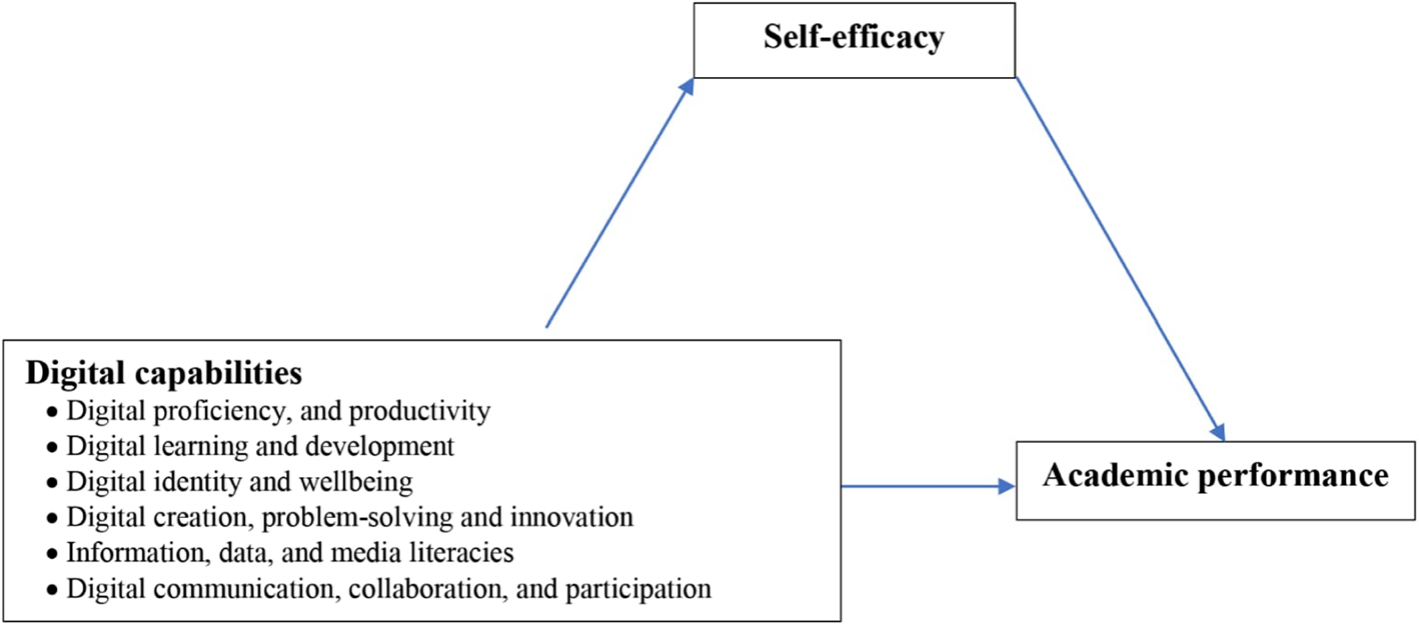
Self-efficacy mediates the reciprocal relationship between digital capabilities and academic performance

Considering these previous findings, it would be beneficial to understand the effect of digital capabilities and self-efficacy on academic performance among nursing students. This is especially important when designing courses designed to boost students’ academic performance and empower them to apply their skills and competences effectively. Furthermore, it is hoped that this study will untangle the intricate threads of digital capabilities and self-efficacy, resulting in more effective use of students’ capabilities and an improvement in nursing students’ efficacious activities in the future. Figure 1 depicts the anticipated reciprocal relationship between digital capabilities and academic performance as mediated by students’ self-efficacy. Therefore, present study was conducted to assess digital capabilities and academic performance among nursing students and investigate the mediating role of students’ self-efficacy.

## Materials and methods

### Research design, setting, and sample

This cross-sectional, correlational, descriptive, and quantitative study was conducted at the Nursing Department of Fatima College of Health Sciences, United Arab Emirates, in the academic year of 2022–2023, where the student intake is exclusively female. Nursing students from levels 2–4 at Fatima College of Health Sciences were included in the study population. These students started to be enrolled in the nursing program from level 2. The student’s grade point average (GPA) was used to measure their academic achievement. The number of participating nursing students was calculated using Epi-Info 7 to account for a 5% variation, 95% confidence, and 0.80 power at a 0.5 significance level by considering a 5% nonresponse rate. The final sample size was 200 convenient nursing students who were available and agreed to participate at that time.

### Instruments

Among the collected demographic data were gender, age, marital status, and student level of education. Digital capabilities were assessed using the digital capabilities and self-efficacy (DCSE) scale, which is a structured self-administered questionnaire from the ZENODO repository that was used to collect data [58]. This scale contained 19 items organized into five dimensions: digital independent learning (5 items; *α* = 0.840), digital information/data management (4 items; *α* = 0.857), digital communication and collaboration (6 items; *α* = .0.858), digital creation (2 items; *α* = .0.759), and digital identity (2 items; *α* = .774).

Variables on the digital independent learning subscale include learning resource accessibility, augmenting lecture notes, accessing course information, studying instructional materials given by instructors, and exploring lecture-related web pages. Elements on the digital information/data management list included database search tools, browsing and watching lecture-related videos, completing assignments, and participating in online learning activities. Items on the Digital Communication and Collaboration Subscale included making my own digital materials for the module, talking to my instructors, communicating with classmates, and offering feedback on their work using various online tools. The items featured on Digital Creation” stand for participating in online group projects with other students and keeping my own blog. Finally, the Digital Identity subscale had indicators for online community involvement: participating actively in my professional community.

The participants rated each item on a seven-point scale (1 = not at all, 7 = to a very great extent). The researchers computed the average score for each component and the overall scale score (*α* = 0.936), which was the average of the five dimensions; higher scores indicated a greater degree of digital skills. The total score, which ranges from 19 to 133 points, can be divided into limited capabilities (19–56 points), intermediate capabilities (57–94 points), or acceptable capabilities (95–133 points). The scale was identified to be satisfactory in terms of its concept validity, criteria validity, and internal consistency reliability (0.975).

Pintrich et al. created the Motivated Learning Strategies Questionnaire (MLSQ) in 1993 [59, 60]. The MSLQ consists of 6 motivational and 9 learning strategies subscales. Self-efficacy for learning and performance is one of the six motivational subscales of the MLSQ [59]. The self-efficacy subscale is an 8-item instrument (α 0.955) that measures a student’s perceptions of their talents and capacity to complete a given activity successfully. Using a seven-point Likert scale ranging from (1 = not at all true of me to 7 = very true of me), participants scored each item. Students were instructed to determine which number between 1 and 7 best represents them. This instrument’s validity and reliability have been confirmed, and it has undergone comprehensive validation in multiple investigations [61, 62].

The total score was obtained by adding and averaging the scores of eight items, with potential averages ranging from 8 to 56, with higher scores indicating more self-efficacy. The total score can be regarded as low efficacy level (8–23 points), moderately efficacious (24–39 points), or highly efficacious (40–56 points).

The academic performance of students is determined by their grade point average; a cumulative grade point average is classed as acceptable (2.0–2.74), good (2.75–3.74) very good (3.75–4.49), and Excellent (4.5–5). The statistical scoring system of the digital capabilities was as follows: high digital capabilities: (66.7–100%), moderate digital capabilities: (33.4–66.6%), and low digital capabilities: (0–33.3%).

## Validity and reliability

Cronbach’s alpha coefficient test was used to measure the internal consistency of items to check the reliability of the study tools. Both tools were found to be reliable at a statistical significance level of *P* < .05, with 0.936 for digital capabilities and self-efficacy (DCSE) tool and 0.955 for Self-efficacy for learning and performance tool.

Ten percent of the participants (*n* = 20) from the context specified above participated in the pilot study to evaluate the clarity and utility of the instruments, identify potential obstacles during data collection, and determine the length of time required to complete the tools. The pilot study participants were not included in the study sample.

### Data collection

The survey was sent to all FCHS students through the students’ service center, which has access to all students’ email addresses. In the email, the letter of information that includes the study purpose, benefits, and participants’ rights was attached. Additionally, it was clearly stated that students have the option to take part in the study or not. The survey requires approximately 15 minutes to be completed. Data were collected over a one-month period. There were no missing data because the data collection was completed after the calculated sample size (*n* = 200) was attained. The researcher’s contact information was supplied. All participant queries were addressed and elucidated.

### Ethical considerations

After submitting the study protocol, instrument, and consent to members of the committee, the Research Ethics Committee of Fatima College of Health Sciences, UAE, sanctioned ethical approval for this study [IRB approval number: FECE-03-20-23-NUR-Rasha]. Subjects volunteered to participate after being informed of the precautions implemented to guarantee data confidentiality. The student’s response was 100% anonymous; therefore, no private information was stored. By disseminating the information sheet and consent form, the nursing students who participated in this study were made aware of the nature and goal of the research endeavor.

### Statistical analysis

The data were analyzed using SPSS 23. Descriptive statistics (frequency, means, standard deviations, and percentages) were deployed to quantify demographic variables. Pearson’s coefficient correlation was used to assess the relationship between the variables in the study. To predict self-efficacy scores and student performance in response to digital capabilities, a multiple regression analysis was undertaken. The variables encompassed as independent variable in the multiple regression models was overall digital capabilities and dependent variables were self-efficacy and cumulative grade point average. Self-efficacy was included to investigate its role as a mediator in the relationship between digital capabilities and student performance. The mediating effect of self-efficacy was investigated using the Hayes Process Model 4 macro software, with digital capabilities as the independent variable, student performance as the dependent variable, and self-efficacy as the mediating variable [63].

## Results

The response rate was 100% after tackling every single participant. Sixty-four percent of the research participants were between 20 and 22 years old. The contestants were all female. Regarding marital status, all undergraduates were single. Considerably more than half (*n* = 107; 53.5%) of the student responders were in level 2 (Table 1). According to Table 2, the overall digital capability level was high (65%), with a mean score of 103.3 ± 19.54. In terms of individual subscales, the “ Digital communication and collaboration “ subscale had the highest mean score (31.12 ± 7.93), while the “Digital creation” subscale had the lowest (8.98 ± 3.73). In addition, the overall self-efficacy was high (67.5%), with a mean score of 43.59 ± 10.13. Table 2 reveals that the overall academic performance of students was moderate (50.5%), with a mean score of 2.97 ± 0.54.

**Table 1:** Socio-demographic facets of research participants (*n* = 200)

Socio-demographic characteristics	No.	%
**Age (years)**		
< 20	61	30.5
20–22	128	64
≥ 23	11	5.5
Min – Max	18.0–24.0	
Mean ± SD	20.54 ± 1.28	
Median	21.0	
**Gender**		
Female	200	100
**Marital Status**		
Single	200	100
**Student Level**		
Level 2	107	53.5
Level 3	52	26
Level 4	41	20.5

**Table 2:** Mean scores of Digital capabilities, Academic Performance, and Self Efficacy (*n* = 200)

Study Variables	Mean Score	Low (< 33.3%)		Moderate (33.3 – < 66.6%)		High (≥66.67%)	
	Mean ± SD.	No.	%	No.	%	No.	%
**Digital Capabilities**							
Digital independent learning	29.11 ± 4.74	0	0	34	17	166	83
Digital information/data management	22.33 ± 4.32	3	1.5	53	26.5	144	72
Digital communication and collaboration	31.12 ± 7.93	11	5.5	69	34.5	120	60
Digital creation	8.98 ± 3.73	42	21	68	34	90	45
Digital Identity	11.71 ± 2.22	1	0.5	35	17.5	164	82
**Overall Digital Capabilities**	103.3 ± 19.54	2	1	68	34	130	65
**Overall Self-Efficacy**	43.59 ± 10.13	5	2.5	60	30	135	67.5
**Academic performance**	2.97 ± 0.54	77	38.5	101	50.5	22	11.0

In terms of the correlation analysis in Table 3, a strong, positive, and significant correlation was noted between academic performance and the overall self-efficacy scale r (198) = 0.308, *p* = 0.001. The previously mentioned correlation was detected not only between academic performance and overall digital capabilities r (198) = 0.315, *p* < 0.001 but also with all subscales of digital capabilities, which were digital independent learning r (198) = 0.223, *p* = 0.002, digital information/data management r (198) = 0.249, *p* < 0.001, digital communication and collaboration r (198) = 0.255, *p* < 0.001, digital creation r (198) = 0.181, *p* = 0.010), and digital identity r (198) = 0.166, *p* = 0.019.

**Table 3:** Matrix of Correlation Between the Variables of the Study (*n* = 200)

	Digital Capabilities						Overall Self-Efficacy	Academic performance
	Digital independent learning	Digital information/ data management	Digital communication and collaboration	Digital creation	Digital Identity	Overall		
Digital independent learning								
**r**	1.000	0.752^*^	0.675^*^	0.496^*^	0.453^*^		0.620^*^	0.223^*^
**p**		< 0.001^*^	< 0.001^*^	< 0.001^*^	< 0.001^*^	< 0.001^*^	< 0.001^*^	0.002^*^
Digital information/data management								
**r**		1.000	0.795^*^	0.589^*^	0.509^*^	0.896^*^	0.522^*^	0.249^*^
**p**			< 0.001^*^	< 0.001^*^	< 0.001^*^	< 0.001^*^	< 0.001^*^	< 0.001^*^
Digital communication and collaboration								
**r**			1.000	0.771^*^	0.463^*^	0.945^*^	0.587^*^	0.255^*^
**p**				< 0.001^*^	< 0.001^*^	< 0.001^*^	< 0.001^*^	< 0.001^*^
Digital creation								
**r**				1.000	0.338^*^	0.792^*^	0.525^*^	0.181^*^
**p**					< 0.001^*^	< 0.001^*^	< 0.001^*^	0.010^*^
Digital Identity								
**r**					1.000	0.588^*^	0.261^*^	0.166^*^
**p**						< 0.001^*^	< 0.001^*^	0.019^*^
Overall Digital Capabilities								
**r**						1.000	0.634^*^	0.315*
**p**							< 0.001^*^	< 0.001*
Overall Self-Efficacy								
**r**							1.000	0.308
**p**								0.001^*^
Academic performance								
**r**								1.000
**p**								

r: Pearson coefficient

*: Statistically significant at *p* ≤ 0.05

Not only was there a substantial positive correlation between the self-efficacy scale and overall digital capabilities r (198) = 0.634, *p* < 0.001, but there was also a substantial positive correlation between the self-efficacy scale and all subscales of digital capabilities, which were digital independent learning r (198) = 0.620, *p* < 0.001, digital information/data management r (198) = 0.522, *p* < 0.001, digital communication and collaboration r (198) = 0.587, *p* < 0.001, digital creation r (198) = 0.525, *p* < 0.001, and digital identity r (198) = 0.261, *p* < 0.001.

To validate the relationship between digital capabilities and self-efficacy, a regression analysis was performed, with digital capabilities as the independent variable and self-efficacy as the dependent variable (Table 4). According to the regression analysis, students’ perception of their digital capabilities could predict their self-efficacy (F (1, 198) = 132.757, *p* < .001, adj. R2 = 0.401).

**Table 4:** Linear regression analysis for Overall Self-Efficacy Scale

	B	Beta	df	t	p	95% CI	
						LL	UL
**Overall digital capabilities**	0.78	0.634	1198	11.522^*^	< 0.001^*^	0.646	0.913
***R***^***2***^ = 0.401, *F* = 132.757^*^, *p* < 0.001^*^							

F, p: f and *p* values for the model

R^2^: Coefficient of determination

B: Unstandardized Coefficients

Beta: Standardized Coefficients

t: t-test of significance

CI Confidence interval, *LL* Lower limit, *UL* Upper Limit

*: Statistically significant at *p* ≤ 0.05

To validate the relationship between digital capabilities and academic performance, a regression analysis was performed, with digital capabilities as the independent variable and academic performance as the dependent variable (Table 5). According to the regression analysis, students’ perception of their digital capabilities could predict their academic performance (F (1, 198) = 15.049, *p* < .001, adj. R2 = 0.071).

**Table 5:** Linear regression analysis for Academic Performance

	B	Beta	df	t	p	95% CI	
						LL	UL
**Overall digital capabilities**	0.008	0.266	1198	3.879*	< 0.001*	0.004	0.013
***R***^***2***^ = 0.071, *F* = 15.049^*^, *p* < 0.001^*^							

F, p: f and *p* values for the model

R^2^: Coefficient of determination

B: Unstandardized Coefficients

Beta: Standardized Coefficients

t: t-test of significance

*CI* Confidence interval, *LL* Lower limit, *UL* Upper Limit

*: Statistically significant at *p* ≤ 0.05

**Fig. 2:**
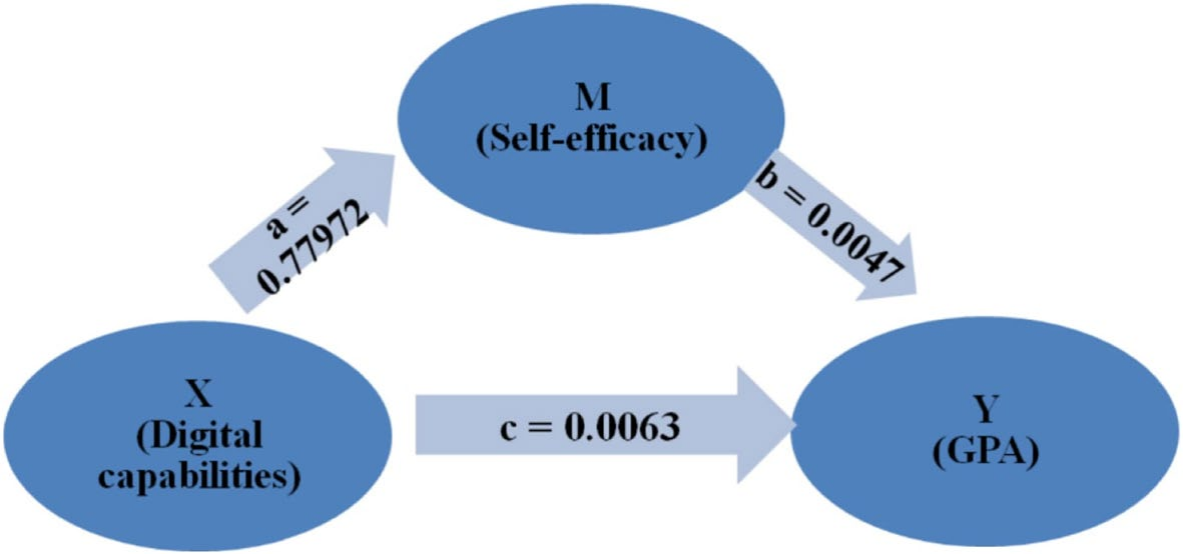
Direct and Indirect effect of Digital Capabilities and Self Efficacy on Students GPA

The study assessed the mediating role of self-efficacy on the relationship between digital capabilities and student performance (Table 6). The results revealed a significant indirect effect of the impact of digital capabilities on student performance (*b* = 0.0036, *t* = 2). Furthermore, the direct effect of digital capabilities on student performance in the presence of the mediator was also found to be significant (*b* = 0.0063, *p* = 0.022). Hence, self-efficacy partially mediated the relationship between digital capabilities and student performance. This means that in the model (Fig. 2), there is a significant positive correlation in paths a–c.

**Table 6:** Path analysis of direct and indirect effects of digital capabilities on students’ performance mediated by self-efficacy

Relationship	Total	Direct Effect	Indirect Effect	95% CI		t–statistics	Conclusion
	Effect			LL	UL		
**Digital capabilities –> Self-Efficacy –> GPA**	0.0100^*^	0.0063^*^	0.0036*	0.0002	0.0075	2	Partial complementary Mediation
	(0.011^*^)	(0.022^*^)				(> 1.96)	
						(sig < 0.05)	

*LL* Lower limit, *UL* Upper Limit

*: Statistically significant at *p* ≤ 0.05

## Discussion

This study investigated how self-efficacy mediates the relationship between nursing students’ digital capabilities and their academic performance (GPA). First, the study revealed that digital capabilities positively affect nursing students’ academic performance. These findings align with several studies that aimed to study the relationship between digital capabilities and academic performance. For example, a recent study by Chen et al. [41] investigated the relationship between digital competence and academic performance among nursing students in China. The study found that nursing students with higher levels of digital competence had better academic performance, including higher exam scores and better grades [41]. These results coincided with those presented among nursing students in Saudi Arabia and Jordan, who found that nursing students with higher levels of digital literacy had better academic performance and better scores on clinical evaluations [48, 64]. Recent studies in South Korea and Pakistan have examined the impact of a digital nursing education program on academic performance among nursing students. These studies found that nursing students who participated in the program had significantly better academic performance, including higher GPAs and better scores on clinical evaluations [37, 65, 66]. On the basis of these findings, it is critical for educational institutions to equip students with suitable technological infrastructures and digital resources to connect their learning with digital learning environments, resulting in good digital education. For nursing students to succeed in healthcare facilities, there is a demand to be involved earlier in the digital world [2].

Second, the current study revealed that self-efficacy had a statistically significant positive correlation with nursing students’ academic performance. Moreover, it had a mediating effect on the relationship between digital capabilities and nursing students’ academic performance. Academic achievement increased among students who reported high levels of self-efficacy. Self-efficacy is described as a person’s confidence in their own ability to plan and carry out a series of activities to achieve a desired result [33]. It has been investigated as a possible factor in integrating theory and practice [37]. The significant relationship between study variables confirms the literature’s claims that these concepts may play a vital role in overcoming the barriers of nursing education.

These findings were consistent with many studies conducted to assess the relationship between self-efficacy and academic performance [67–69]. In a systematic review that analyzed 12 years of research on the relationship between academic self-efficacy and the academic performance of university students, researchers determined that fifty-nine studies found a moderate correlation between academic self-efficacy and academic performance [70]. In contrast, a study was conducted to evaluate self-efficacy on students’ performance among nursing students. The results of the 296 participants’ study concluded that there is no relationship between self-efficacy and students’ performance, which means that students with high self-efficacy do not necessarily improve students’ performance in clinical settings [71].

Last, given the widespread incorporation of technological advances in the field of education, the acquisition of digital self-efficacy is deemed an essential competency. Put differently, digital self-efficacy enables students to obtain access to unlimited educational resources and enhances their interaction in the learning environment. Recent researchers found that nursing students with high levels of technologic self-efficacy were more likely to apply it in their academic work [67–69].

Hence, self-efficacy reinforcement may be an effective way to foster nursing students’ digital capabilities and prepare them for the increasingly digital healthcare environment. These results suggested including digital technologies and self-efficacy in nursing education by identifying factors to improve their digital capabilities in a tech environment. It is necessary to develop a strategy to improve nursing students’ digital capabilities and self-efficacy.

## Implications and limitations

The study’s findings have significant consequences for nursing educators, legislators, and students. To improve students’ digital capabilities and overall performance, instructors should assess their curriculum and thoroughly think about incorporating digital technologies into the designs they produce. Implementing novel approaches to teaching, expanding access to a broader range of information and resources, and developing new skills for the digital era are all possibilities to improve the pedagogical system. Guidelines should specifically recognize the promise for digital capabilities to boost student outcomes. Enhancing nursing students’ digital capabilities can be a useful approach for improving both academic achievement and self-efficacy. The relationship between self-efficacy and academic success has implications for practice.

According to the research, nursing students should strive to develop their self-efficacy throughout their undergraduate careers since it predicts their success in the workforce after graduation. They must also dedicate time to their education and strive to improve themselves. According to the study’s findings, digital capabilities and self-efficacy were extremely advantageous in the curriculum, which could give rise to greater job possibilities.

Although the study provides fresh viewpoints for theory and future investigations, it does have limitations. An online survey of undergraduate nursing students was used in the study. This may reduce the findings’ applicability in other settings. Another limitation is the possibility of bias created by self-reporting. In the context of this study, self-report was used to assess digital capabilities, self-efficacy, and self-reported GPAs. Furthermore, because of the wide range of programs, the relatively small number of participants, and the inclusion of only undergraduate nursing students, the results should be cautiously generalized to a broader population.

## Conclusion

Overall, we believe our findings provide a solid foundation for future research on digital competence and self-efficacy. Students who are well-versed and skilled are more capable of absorbing and using what they have learned. Academic advancement and self-confidence in one’s own capacity to succeed are both considered to be rooted in the growth of DC skills among undergraduates. Therefore, digitization is critical to the evolution of any modern civilization, as a shortage of competent employees would hinder society’s future development. Based on the findings of this study, we can conclude that performance prediction will benefit educators and the educational institution as a whole by allowing them to modify their pedagogical techniques and assist students in optimizing their own learning strategies. Students with a low level of self-efficacy and limited digital capabilities should be looked at frequently and provided with further support to reduce the probability that they will drop prematurely from college.

## Data Availability

The dataset gathered and analyzed in the current work is not accessible to the general public; however, it is obtainable from the corresponding author given adequate justification upon request.

## Abbreviations

CGPA: Cumulative Grade Point Average
JISC: Joint Information System Committee
ASE: Academic Self-Efficacy
DCSE: Digital Capabilities and Self-efficacy
